# Super-resolution microscopy reveals ultra-low CD19 expression on myeloma cells that triggers elimination by CD19 CAR-T

**DOI:** 10.1038/s41467-019-10948-w

**Published:** 2019-07-17

**Authors:** Thomas Nerreter, Sebastian Letschert, Ralph Götz, Sören Doose, Sophia Danhof, Hermann Einsele, Markus Sauer, Michael Hudecek

**Affiliations:** 1 Medizinische Klinik und Poliklinik II, Universitätsklinikum Würzburg, Oberdürrbacherstrasse 6, 97080 Würzburg, Germany; 2 Department of Biotechnology and Biophysics, Biocenter, Julius Maximilian University Würzburg, Am Hubland, 97074 Würzburg, Germany

**Keywords:** Cancer imaging, Cancer immunotherapy, Imaging

## Abstract

Immunotherapy with chimeric antigen receptor-engineered T-cells (CAR-T) is under investigation in multiple myeloma. There are reports of myeloma remission after CD19 CAR-T therapy, although CD19 is hardly detectable on myeloma cells by flow cytometry (FC). We apply single molecule-sensitive direct stochastic optical reconstruction microscopy (*d*STORM), and demonstrate CD19 expression on a fraction of myeloma cells (10.3–80%) in 10 out of 14 patients (density: 13–5,000 molecules per cell). In contrast, FC detects CD19 in only 2 of these 10 patients, on a smaller fraction of cells. Treatment with CD19 CAR-T in vitro results in elimination of CD19-positive myeloma cells, including those with <100 CD19 molecules per cell. Similar data are obtained by *d*STORM analyses of CD20 expression on myeloma cells and CD20 CAR-T. These data establish a sensitivity threshold for CAR-T and illustrate how super-resolution microscopy can guide patient selection in immunotherapy to exploit ultra-low density antigens.

## Abstract (web summary)

CD19 CAR-T cells have achieved some success in treating myeloma patients despite the limited detection of the CD19 antigen. Here, the authors show using *d*STORM that 10/14 myeloma samples studied express ultra-low levels of CD19, which are sufficient for engaging CAR-T cells in vitro.

## Introduction

The B-lymphocyte antigen CD19 is pursued as a target in adoptive immunotherapy with T-cells that are gene-engineered with a CD19-specific chimeric antigen receptor (CD19 CAR-T). CD19 CAR-T have recently been approved as a potentially curative treatment for patients with relapsed/refractory B-cell acute lymphoblastic leukemia and non-Hodgkin’s lymphoma^1–4^. In these diseases, CD19 is nearly uniformly expressed on malignant cells, with an antigen density in the order of several thousands of molecules per cell^1,2,5^, which is thought to be an optimal range for recognition by CD19 CAR-T. In contrast, CD19 is considered a rarely and infrequently expressed target antigen in other hematologic malignancies, including multiple myeloma, which is characterized by clonal proliferation of plasma cells that produce aberrant immunoglobulin^6,7^. Despite aggressive treatment with polychemotherapy, myeloma remains incurable in the majority of patients^8^.

A recent study reported on the clinical efficacy of CD19 CAR-T in heavily pre-treated myeloma patients with one complete (CR) and several partial responses (PR) after myeloablative chemotherapy and autologous hematopoietic stem cell transplantation (HSCT) followed by CD19 CAR-T administration^6,9^. The achievement of CR was attributed to the administration of CD19 CAR-T even though, according to conventional detection by flow cytometry (FC), CD19 was only present on a minute fraction of myeloma cells (0.05% by FC) in this patient^6^. This sparked heavy controversy over the antigen-sensitivity of CD19 CAR-T, and the ability of FC to detect ultra-low CD19 expression.

In previous work, we have demonstrated the capacity of direct stochastic optical reconstruction microscopy (*d*STORM) to determine absolute copy numbers of molecules on plasma membranes of human cells^10,11^. *d*STORM exhibits single-molecule sensitivity^12,13^, suggesting that it could be used to detect ultra-low expression of CD19 on myeloma cells that is undetectable by FC. We hypothesized that CD19 may be expressed on a proportion of myeloma cells at a molecular density that is below the detection limit of FC but that is sufficient for recognition and elimination by CD19 CAR-T. To test this hypothesis, we used *d*STORM to generate expression profiles of CD19 on myeloma cells and to assess elimination of CD19-positive myeloma cells by CD19 CAR-T in vitro. We show that CD19 is indeed expressed on a substantial fraction of myeloma cells at ultra-low density in the majority of patients, and demonstrate that <100 CD19 molecules per myeloma cell trigger elimination by CD19 CAR-T. Analysis by *d*STORM is more sensitive compared to FC in detecting CD19, and accurately predicts elimination of CD19-positive myeloma cells by CD19 CAR-T. Our findings are corroborated by *d*STORM analyses of CD20 expression and data showing elimination of CD20-positive myeloma cells by CD20 CAR-T with similar sensitivity.

## Results

### Patient cohort and CD19 expression on myeloma cells by FC

**Fig. 1:**
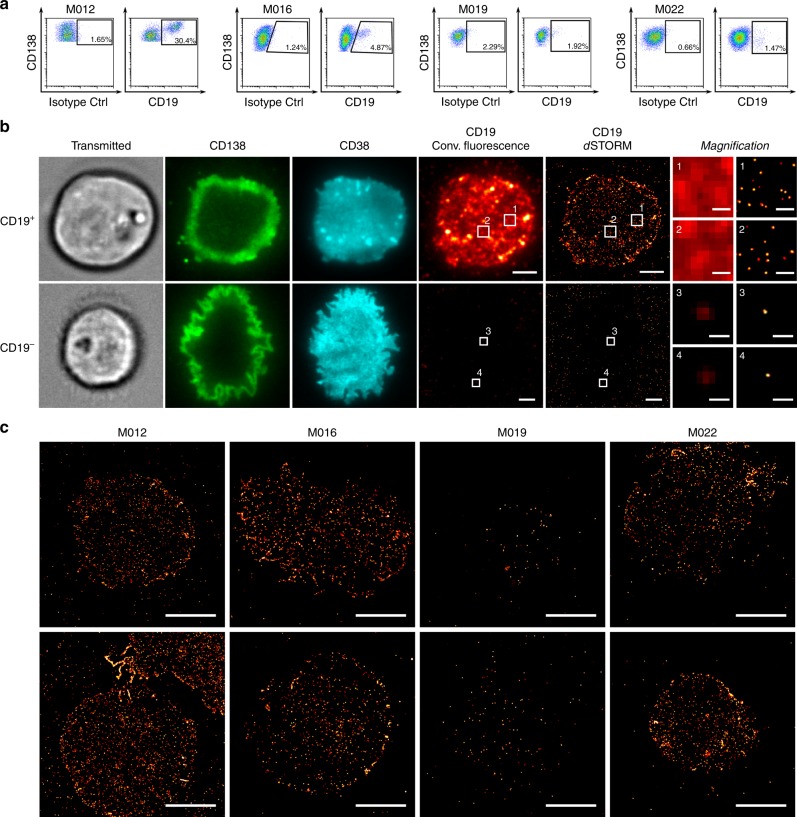
Detection of CD19 on myeloma cells using flow cytometry and *d*STORM. **a** Flow cytometric analysis of CD19 expression on primary myeloma cells purified from bone marrow aspirates. Gating strategy for dot plots shown: FSC/SSC plasma cell gate → 7-AAD^−^ → CD138^+^/CD38^+^. Plots show expression of CD19 or isotype control (Ctrl, *x-*axis) vs. CD138 (*y-*axis). Exemplary patients where myeloma cells comprised a CD19-positive fraction by FC (patient M012 & patient M016), were CD19-negative (patient M019) or showed an ambiguous expression (patient M022). Data for all patients are shown in Table 1 and Supplementary Fig. 1. **b** Detection of CD19 on multiple myeloma cells using *d*STORM. Myeloma cells were identified by transmitted light microscopy and expression of CD138 and CD38 as detected by conventional wide-field fluorescence microscopy. CD19 was detected on primary myeloma cells using conventional wide-field fluorescence and *d*STORM. Images depict CD19 molecules in the bottom plasma membrane (attached to glass surface) of a CD19-positive (top row) and a CD19-negative myeloma cell (bottom row). Small panels display magnification of boxed regions revealing the markedly enhanced sensitivity of *d*STORM. **c** Exemplary images of CD19-positive cells as detected by *d*STORM. Myeloma cells were identified as stated in **b** and CD19 was detected on primary myeloma cells using *d*STORM for patients M012 (top image: 3.5 CD19/µm^2^; bottom image: 4.9 CD19/µm^2^), M016 (top image: 3.3 CD19/µm^2^; bottom image: 1.8 CD19/µm^2^, M019 (top image: 0.16 CD19/µm^2^; bottom image: 0.21 CD19/µm^2^ and M022 (top image: 3.6 CD19/µm^2^; bottom image: 5.2 CD19/µm^2^. The representative images depict CD19 molecules in the basal plasma membrane. Scale bars, 3 µm and 0.4 µm (magnification) (**b**), 5 µm (**c**)

To generate expression profiles of CD19 on primary myeloma cells by FC and *d*STORM, we obtained bone marrow from *n* = 14 consecutive patients with multiple myeloma that had measurable disease by histopathology. In this patient series, *n* = 4 patients had newly diagnosed myeloma, and *n* = 10 patients had been previously treated and were either in a state of partial remission (*n* = 2) or had progressing disease (*n* = 8) (Supplementary Table 1). First, we performed FC to detect CD19 on myeloma cells. In two of the 14 patients (M012 and M016), we found a clearly distinguishable CD19-positive myeloma cell population, comprising 30.4% and 4.9% of cells, respectively (Fig. 1a). In the remaining 12 patients, myeloma cells were either CD19-negative or contained only a minute population of myeloma cells (<3%) in which the signal obtained after staining for CD19 could not be clearly discriminated from background (Fig. 1a and Supplementary Fig. 1).

### *d*STORM is more sensitive than FC in detecting CD19

To establish the detection limit and sensitivity threshold of *d*STORM and FC, we performed antibody titration experiments on the human leukemia cell line NALM-6, which uniformly expresses CD19 (phenotype: CD19^+^CD38^+^CD138^+^). The specificity of the anti-CD19 antibody used for *d*STORM and FC was confirmed by staining CD19-negative (K562) and CD19-positive cell lines (K562_CD19, NALM-6, Supplementary Fig. 2). NALM-6 cells were stained with serial dilutions of anti-CD19 mAb (10 µg/ml–50 pg/ml) (Supplementary Fig. 3A). Under saturating conditions (*c* ≥ 2.5 μg/ml), we detected 3.4 ± 0.2 (SEM, standard error of the mean) CD19 molecules/µm^2^ by *d*STORM, which corresponds to 1780 ± 210 CD19 molecules per NALM-6 cell. With each dilution, we obtained gradually lower numbers of CD19 molecules (Supplementary Table 2). The detection limit of *d*STORM was 0.006 ± 0.002 CD19 molecules/µm^2^, which corresponds to 3.1 ± 1.3 CD19 molecules per cell (*c* = 50 pg/ml) (Supplementary Fig. 3B–D). At all concentrations, *d*STORM correctly classified all NALM-6 cells as being CD19-positive. By FC, uniform CD19 expression on NALM-6 cells was only detectable when the anti-CD19 mAb was used at c ≥ 50 ng/ml (Supplementary Fig. 3A, B), suggesting a 3-log difference in sensitivity between *d*STORM and FC. Taken together, these data show that *d*STORM is more sensitive than FC in detecting CD19, and able to visualize CD19 molecules on tumor cells with single-molecule resolution.

### Expression profile of CD19 on myeloma cells by *d*STORM

We hypothesized, that in addition to CD19^high^ myeloma cells that are readily detectable by FC, there is an as-yet undetected population of CD19^low^ myeloma cells that is invisible to FC but can be visualized by *d*STORM. To test this, we attempted FC-based cell sorting to separate CD19-positive and CD19-negative myeloma cells but found that the number of cells that survived this procedure was insufficient to perform subsequent *d*STORM-analyses.

Therefore, we applied *d*STORM analysis to the same samples of myeloma cells of all patients (*n* = 14) that had been examined by FC. We identified myeloma cells by their expression of CD38 and CD138 using fluorescence microscopy, and CD19 expression was evaluated by *d*STORM with single-molecule resolution (Fig. 1b). Thereby, we were able to detect CD19-positive myeloma cells in 10 out of the 14 patients. These included the *n* = 2 patients that were positive by FC as well as eight additional patients that had been classified as CD19-negative by FC. In 4 out of the 14 patients, no CD19-positive myeloma cells were detectable (Fig. 1c and Supplementary Fig. 4).

### *d*STORM detects more CD19-positive myeloma cells than FC

**Fig. 2:**
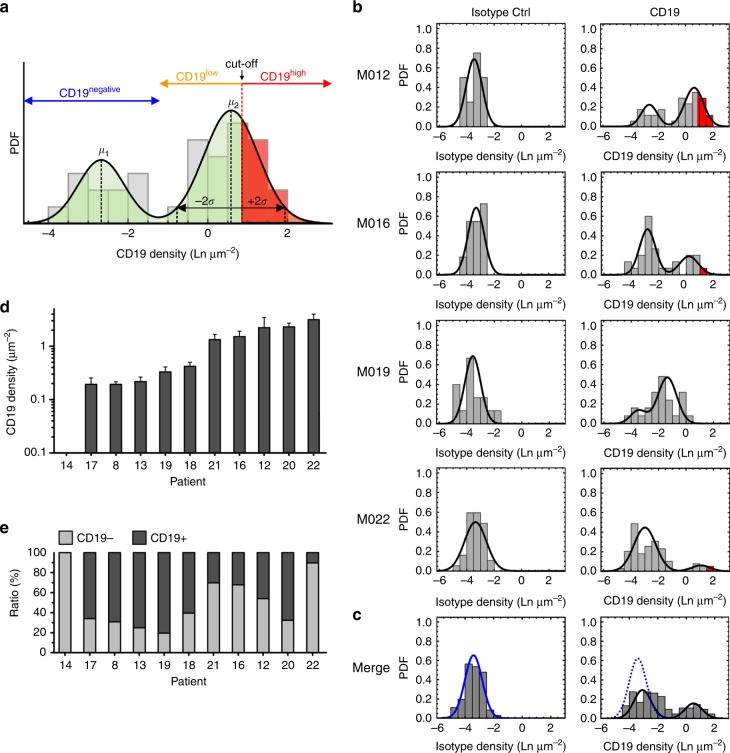
CD19 expression profiles on myeloma cells established by *d*STORM. **a** Schematic expression profile of CD19. Density distributions were divided into a CD19-positive subpopulation (CD19-positive cells) and a CD19-negative subpopulation (CD19-negative cells; blue range). The latter group was defined by the density distribution pattern of the isotype control antibody (non-specific binding of the control antibody to the plasma membrane and glass surface). Distributions were fitted to a two-component log-normal function with mean (*µ*) and standard deviation (*σ*), to calculate density ranges from small (*µ* − 2*σ*) to large (*µ* + 2*σ*) values. The CD19-positive population was further divided into a CD19^low^ (orange range) and a CD19^high^ subpopulation (red range), based on the cutoff value of 1350 molecules per cell (see text for further details). **b**, **c** Expression profiles for CD19 on myeloma cells from patient M012, M016, M019, M022 and a merge **c** of these four patients generated by *d*STORM. Distribution plots show the relative CD19 density obtained after staining with isotype control (left column) and anti-CD19 antibody (right column) on myeloma cells. Densities are provided as logarithmic numbers (natural logarithm, Ln) of molecules per µm^2^. Probability density functions (PDFs) were fitted with a one or two-component log-normal function that was dependent on the fit-accuracy calculated with an Anderson-Darling test (rejected at a *p*-value < 0.05). For each patient, the percentage of myeloma cells that had been determined to be CD19-positive by flow cytometry is provided for comparison (red segments). Dotted blue line in merge panel: function of the isotype control. Data for all patients are shown in Table 1 and Supplementary Fig. 4. **d** Mean CD19 molecule density on CD19-positive myeloma cells in molecules per µm^2^ as assessed by *d*STORM imaging in *n* = 10 CD19-positive myeloma patients, and one CD19-negative patient (M014) for comparison. Error bars represent standard deviations of the means from repeated random sampling of the estimated log-normal distribution. **e** Percentages of CD19-positive and CD19-negative cells, ranging from 10% (M022) to 80% (M019) of CD19-positive cells among patients

First, we focused on the evaluation of patients M012 and M016 that had been correctly identified as being CD19-positive by FC. We generated CD19 density plots from myeloma cells of these patients, showing a clear segregation into CD19-positive and CD19-negative myeloma cells (patient M012, Fig. 2a, b). In both patients, the percentage of myeloma cells on which we detected CD19 by *d*STORM was higher compared to FC: in patient M012 this were 68% by *d*STORM vs. 30.4% by FC, and in patient M016 this were 32% by *d*STORM vs. 4.9% by FC (Fig. 2b and Table 1). The average density of CD19 on all CD19-positive myeloma cells from patient M012 was 1200 ± 580 molecules per cell (Table 1). We reasoned that FC had only detected myeloma cells with the highest CD19 expression and quantified CD19 molecules from cells in the top 30.4% of the density plot (which was the percentage of CD19-positive myeloma cells by FC) (Fig. 2a). We found that the average number of CD19 molecules on these CD19^high^ myeloma cells was 2240 ± 260 molecules per cell compared with 750 ± 60 molecules in the remaining, CD19^low^ myeloma cells (Table 1). The cutoff value separating CD19^high^ and CD19^low^ myeloma cells at the 30.4th percentile of the density plot was 1350 CD19 molecules per cell. We obtained similar data for patient M016 (Table 1 and Fig. 2b).

**Table 1:** Summary of data obtained by *d*STORM and flow cytometry

#	ID	Flow Cytometry Δ% CD19^+^ (% anti-CD19 - % isotype)	*d*STORM % CD19^+^	No of CD19^+^ cells analyzed by *d*STORM	CD19 molecules/ cell^a^ CD19^+^ (range)	CD19 molecules/ cell^a^ CD19^low^	CD19 molecules/ cell^a^ CD19^high^	Elimination by CD19 CAR-T	% IFNγ producing CD19 CAR-T
1	M007	0 (0.1–0.1)	0	–	N/A	N/A	N/A	N/A	0
2	M008	0 (1.2–0.9)	69.2	65	110 (22–340)	110	0	+	4.9
3	M011	(s) (0.8–0.6)	0	–	N/A	N/A	N/A	N/A	0
4	M012	29 (30.4–1.6)	67.6	34	1200 (250–3700)	750	2240 (30%)	+	8.9
5	M013	0.9 (1.2–0.3)	75.1	45	93 (19–290)	93	0	+	0.3
6	M014	1.7 (4.0–2.3)	0	–	N/A	N/A	N/A	N/A	0
7	M015	(s) (1.1–1.3)	0	–	N/A	N/A	N/A	N/A	0
8	M016	3.7 (4.9–1.2)	32.1	31	530 (110–1650)	470	1850 (4%)	+	1.2
9	M017	0 (0.7–1.4)	66.0	20	64 (13–200)	64	0	+	0.8
10	M018	0 (0.4–1.7)	60.4	33	270 (55–830)	270	0	+	0
11	M019	0 (1.9–2.3)	80.3	25	140 (28–420)	140	0	+	0.4
12	M020	2.4 (5.7–2.3)	46.0	36	950 (200–3000)	680	2090 (19%)	+	ndt
13	M021	1.3 (3.1–1.8)	30.2	38	630 (130–2000)	530	1900 (7%)	+	0
14	M022	0.8 (1.5–0.7)	10.3	80	1600 (330–5000)	830	2500 (47%)	+	0

(s): single events

*ndt* cytokine production was not assessed for patient M020

^a^Mean values, in brackets: Calculated data ranging from small (exp (µ−2*σ*)) to high (exp (µ+2*σ*)) values (95.45% of all values lie within this range). CD19-positive cells with >1350 molecules per cell were classified as CD19^high^ and were otherwise classified as CD19^low^ (simulated data). Δ% CD19^+^: the percentage of the cells in the CD19-positive gate for the isotype control was subtracted from the percentage of cells in the CD19-positive gate for the respective CD19 staining

### *d*STORM detects CD19 in patients judged CD19-negative by FC

Next, we focused on the eight patients who were classified as CD19-positive by *d*STORM and as CD19-negative by FC. In these patients, we detected CD19-positive myeloma cells by *d*STORM with varying levels of expression (mean values 64–1600 molecules per cell, Fig. 2c–e and Supplementary Fig. 4). In five of these eight patients, myeloma cells were exclusively CD19^low^ (mean values 64–270 molecules per cell). In the remaining three of these eight patients, we also detected a proportion of myeloma cells with CD19^high^ expression (mean values 1900–2500 molecules per cell) (Table 1). In total, the CD19-positive cells comprised between 10.3 and 80.3% of the entire myeloma cell population (mean: 54 ± 7%; of all 10 CD19-positive myeloma patients) (Fig. 2e and Table 1). Taken together, our data show that CD19 is expressed at low and ultra-low levels on a substantial proportion of myeloma cells in patients that are falsely classified as being CD19-negative by FC.

### CD19^low^ myeloma cells are eliminated by CD19 CAR-T

**Fig. 3:**
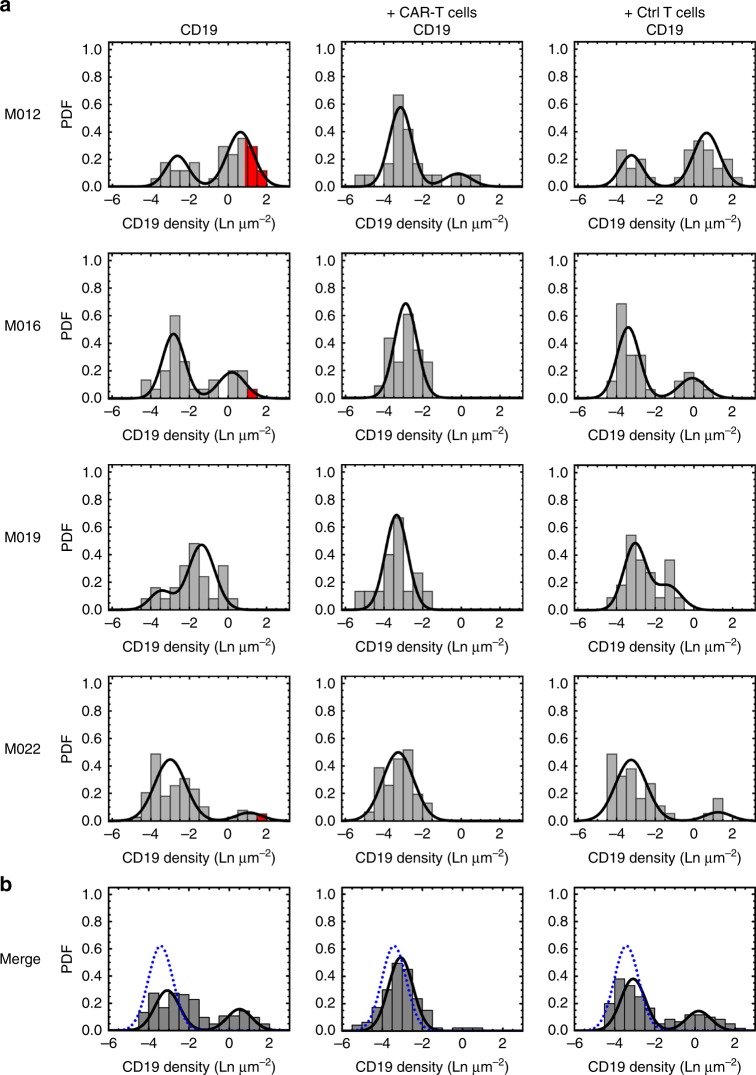
Elimination of CD19-positive myeloma cells by CD19 CAR-T. **a** Expression profiles for CD19 on myeloma cells from patient M012, M016, M019, M022 and a merge of these four patients (**b**) generated by *d*STORM. Distribution plots showing the relative CD19 density obtained after staining with anti-CD19 antibody on myeloma cells before and after treatment with CD19 CAR-T or untransduced control T cells. Densities are provided as logarithmic numbers (natural logarithm, Ln) of molecules per µm^2^. Probability density functions (PDFs) were fitted with a one or two-component log-normal function that was dependent on the fit-accuracy calculated with an Anderson-Darling test (rejected at a *p*-value < 0.05). For each patient, the percentage of myeloma cells that had been determined to be CD19-positive by flow cytometry is provided for comparison (red segments). For better visualization, the function of the isotype control is included in the CD19 staining for the merge panels (dotted blue line). Data for all patients are shown in Table 1 and Supplementary Fig. 4

To investigate whether CD19 expression on CD19^high^ and CD19^low^ myeloma cells is sufficient for CAR-T recognition, we treated them with CD19 CAR-T for 4 h in vitro and then repeated the *d*STORM-analysis. In all patients that contained CD19^high^ and CD19^low^ myeloma cells, we found that CD19-expressing myeloma cells were eliminated and only CD19-negative myeloma cells were present after the treatment (Fig. 3 and Supplementary Fig. 4). Control T-cells derived from the same donor and not equipped with the CD19 CAR did not confer any relevant reactivity against CD19^high^ and CD19^low^ myeloma cells (Fig. 3 and Supplementary Fig. 4). The complete elimination of CD19^low^ myeloma cells indicated that CD19 CAR-T required an antigen density of <100 CD19 molecules to recognize and eliminate a myeloma cell. To exclude the potential that elimination of CD19^low^ myeloma cells had occurred due to bystander killing (i.e., due to cytolytic granules released from CD19 CAR-T after being triggered by CD19^high^ myeloma cells), we repeated the CD19 CAR-T treatment assay with myeloma cells that were exclusively CD19^low^. In all patients, we found, that CD19 CAR-T eliminated CD19^low^ myeloma cells, including CD19^low^ myeloma cells from patients M017 and M013, that expressed on average 64 ± 8 and 93 ± 10 CD19 molecules per cell, respectively (Supplementary Fig. 4). Collectively, these data demonstrate that CD19 CAR-T are capable of rapidly eliminating myeloma cells that express ultra-low levels of CD19 (Fig. 3 and Supplementary Figs. 4 and 5). Further, the data establish the antigen threshold required for triggering the cytolytic activity of CD19 CAR-T, which is below 100 CD19 molecules per target cell.

### IFNγ release by CAR-T does not predict CD19 expression

We sought to determine whether intracellular staining for IFNγ production in CD19 CAR-T after coculture with myeloma cells could be used as a surrogate assay to test for the presence of CD19^low^ myeloma cells instead of *d*STORM microscopy. However, the IFNγ assay only worked in two of the ten patients that we had shown to contain CD19-positive myeloma cells by FC and *d*STORM (Supplementary Fig. 6). These data suggest that the antigen threshold required for inducing cytokine production in CD19 CAR-T is higher compared to the threshold required for inducing cytolytic activity, consistent with prior reports^14^. In summary, these data show that conventional detection (by FC) and analytical methods (by IFNγ secretion assay) are not sensitive enough to reveal CD19^low^ expression on myeloma cells.

### CD20 CAR-T eliminate CD20^low^ myeloma cells

**Fig. 4:**
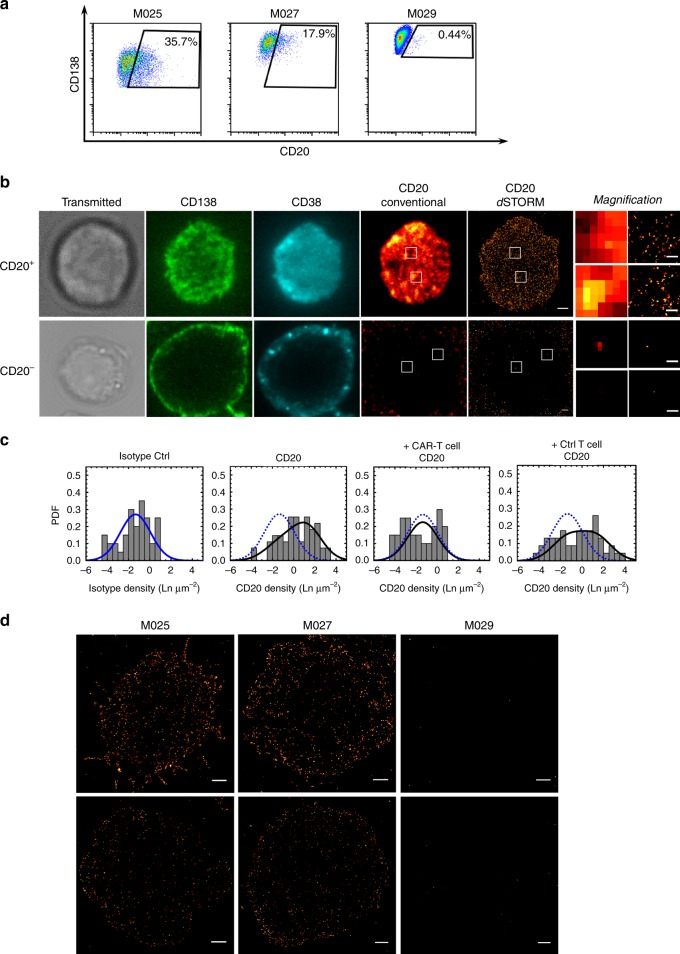
Detection of CD20-positive myeloma cells by flow cytometry and *d*STORM and elimination by CD20 CAR-T. **a** Flow cytometric analysis of CD20 expression on primary myeloma cells purified from bone marrow aspirates. Gating strategy: FSC/SSC plasma cell gate → 7-AAD^−^ → CD138^+^/CD38^+^. Patients with myeloma cells comprising a CD20-positive fraction by FC (patient M025 & patient M027), or being CD20-negative (patient M029) are shown. Data for all patients are shown in Table 3. **b** Detection of CD20 using *d*STORM. Myeloma cells were identified by transmitted light microscopy and expression of CD138 and CD38 as detected by conventional wide-field fluorescence microscopy. CD20 was detected on primary myeloma cells using conventional wide-field fluorescence and *d*STORM. Images depict CD20 molecules in the bottom plasma membrane (attached to glass surface) of a CD20-positive (top row) and a CD20^−^ myeloma cell (bottom row). Small panels display magnification of boxed regions revealing the markedly enhanced sensitivity of *d*STORM. **c** Merged expression profiles for CD20 on myeloma cells from patients M025, M027, & M029 generated by *d*STORM. Far left and left column: relative CD20 density obtained after staining with isotype control (far left column) and anti-CD20 antibody (left column). Densities are provided as logarithmic numbers (natural logarithm, Ln) of molecules per µm^2^. Density plots were divided into CD20-positive and CD20-negative subpopulations, defined by the density distribution pattern of the isotype control antibody. Density plots were fitted with a one or two-component log-normal function. Right and far right column: relative CD20 density obtained after staining with anti-CD20 antibody on myeloma cells after treatment with CD20 CAR-T (right column) or untransduced control T cells (far right column). Dotted blue line: function of the isotype control. Data for single patients are shown in Supplemementary Table 3 and Supplementary Fig. 8. **d** Exemplary images of CD20-positive cells as detected by *d*STORM. Myeloma cells were identified as stated in **b** and CD19 was detected on primary myeloma cells using *d*STORM for patients M025, M027, and M029. The representative images depict CD20 molecules in the bottom plasma membrane (attached to glass surface). Scale bars, 1 µm and 0.2 µm (magnifications) (**b**), 3 µm (**d**)

To extend our findings beyond CD19, we investigated expression of CD20, another B-lineage antigen usually considered to be absent on myeloma cells^15^. We performed FC analyses of primary myeloma cell samples from three additional patients, and classified one out of these three patients (M029) as being CD20-negative. In the remaining two patients, FC detected a CD20-positive myeloma cell population accounting for 33% (M025) and 16.8% (M027), respectively. (Fig. 4a and Supplementary Fig. 7). By *d*STORM, we detected a CD20-positive myeloma cell population in these two patients as well however, the percentage of CD20-positive myeloma cells was substantially greater compared to FC (76.7% vs. 33%, M025 and 64.7% vs. 16.8%, M027). Subsequent calculation of the antigen density on the myeloma cell surface resulted in mean values of 650–1770 CD20 molecules per cell. A 4-h coculture with CD20 CAR-T led to the complete eradication of CD20-expressing myeloma cells in these patients, showing that the exquisite antigen-sensitivity of CAR-T is not limited to CD19, but also applies to other target antigens (Fig. 4b–d, Supplementary Fig. 8, and Supplementary Table 3).

## Discussion

CD19 is under investigation as target antigen for CAR-T immunotherapy in multiple myeloma. A recent study reported complete remission in a myeloma patient who received CD19 CAR-T after myeloablative chemotherapy and autologous HSCT, even though only a minute fraction of myeloma cells was CD19-positive as assessed by FC^6^. Several additional study patients achieved partial responses or stable disease. With longer follow-up, it has become apparent that these antimyeloma responses were not durable; still the study outcome was unexpected given the advanced stage and the heavy prior treatment in this myeloma patient cohort^9^. Consequently, it has been speculated that CD19 may be expressed on a higher proportion of myeloma cells than can be identified by FC, including myeloma cells that express CD19 at very low levels, which may however, be sufficient for recognition by CD19 CAR-T^6,16,17^. An obstacle to testing this hypothesis is the relative insensitivity of FC, the prevailing detection method in clinical routine, with a detection limit in the order of a few thousands of molecules per cell^5,18,19^. In addition, the precise antigen threshold on tumor cells required for engaging and subsequently activating CAR-T has thus far been unknown. Several studies have attempted to extrapolate the lower detection limit of CAR-T with model cell lines, providing estimates in the range of hundreds of target molecules per cell^14,20^. However, these estimates have not been rigorously verified owing again to the lacking ability of FC to detect such low antigen levels on target cells with confidence.

Here, we applied single-molecule-sensitive super-resolution microscopy by *d*STORM and show that in 10 out of 14 myeloma patients, CD19 is expressed on a considerable fraction of myeloma cells comprising up to 80% of the entire myeloma cell population. We show that in the majority of myeloma cells, the expression level of CD19 is below the detection limit of FC and can only be visualized using *d*STORM. Our data demonstrate that FC underestimates the percentage of myeloma cells that express CD19 and falsely classifies myeloma cells in 8 out of 10 patients as CD19-negative, even though CD19 is expressed on a fraction of myeloma cells at low levels as revealed by *d*STORM imaging. Our data suggest that myeloma cells that express <1350 CD19 molecules cannot be detected by FC, which is consistent with previous reports on the sensitivity of this method in clinical routine^14,18–20^.

Notably, myeloma cells that were judged CD19-negative by FC in the Garfall et al. study^6^ also appeared CD19-negative by quantitative polymerase chain reaction (qPCR). However, depending on the sensitivity threshold and reference gene used for the qPCR analysis, this does not exclude the potential that at least some CD19 transcripts and at least some CD19^dim^ myeloma cells were present in the analysis sample^21^, that could have been visualized using *d*STORM.

We show that in each of the CD19-positive myeloma patients, the subpopulation of CD19-expressing myeloma cells (either at high or low density) were readily eliminated after a short treatment with CD19 CAR-T in vitro. The CD19-CAR employed in our study has been validated to be clinically effective in ALL and NHL^1,2^. Accordingly, our data suggest that CD19 CAR-T may also be effective against CD19-expressing myeloma cells in vivo. However, our data also show that in each of the ten patients, there was a fraction of CD19-negative myeloma cells that were not eliminated by CD19 CAR-T. These data suggest that complete responses of multiple myeloma after CD19 CAR-T therapy may only be accomplished in conjunction with another effective antimyeloma treatment, or a dual-antigen targeting approach where CAR-T concomitantly recognize CD19 in combination with, e.g., B-cell maturation antigen (BCMA) or another myeloma antigen^9^. Indeed, recent studies with CD19 CAR-T in ALL and with BCMA-CAR-T in myeloma have shown that the presence of antigen-negative leukemia or myeloma cells leads to outgrowth of these cells and rapid relapse^22,23^. In patients that experienced CD19-negative ALL-relapse, treatment with CAR-T cells that recognized CD22 as rescue antigen, was able to re-induce remissions in some patients^24^.

CARs are synthetic receptors and even though CD19 CAR-T have accomplished clinical approval in ALL and NHL, their mechanism of action is still a black box at the molecular level. A particular interest has been to determine the antigen-sensitivity of CAR-T, both for predicting efficacy and for assessing safety. Here, we provide evidence that CD19 CAR-T are able to recognize and eliminate myeloma cells that express <100 CD19 molecules on their surface. These data establish the sensitivity threshold for CAR-T cells and surpass predictions that have been made in previous studies with model tumor cell lines^14,20^, but were limited by the inability of FC to enumerate antigens with single-molecule resolution. Our data support the prior notion that CAR-T are more sensitive than conventional antibodies and bi-specific antibodies in detecting surface molecules on tumor cells^14^.

Our data show that ultra-low-level expression of CD19 on myeloma cells is sufficient for recognition and elimination by CD19 CAR-T, suggesting that CD19 CAR-T may also be clinically effective against myeloma cells that express ultra-low levels of antigen. In our in vitro assay, we make two basic assumptions: first, we assume that CD19 CAR-T get access to and are able to engage CD19 molecules on myeloma cells, which is also the case in vivo when CD19 CAR-T migrate through the bone marrow. Second, we assume that the stoichiometry of CD19 CAR-T and myeloma cells is such that the cytolytic activity of CD19 CAR-T leads to a reduction in myeloma cell burden. In a clinical setting, this would either be accomplished by administering a high dose of CD19 CAR-T, or through in vivo proliferation and expansion of CD19 CAR-T after infusion. Our data show that ultra-low-level expression of CD19 (i.e., <100 molecules per target cell) is sufficient for inducing cytolytic activity of CD19 CAR-T, but insufficient for inducing cytokine secretion and proliferation, in accordance with previous investigations^20,25^. These data are consistent with the clinical observation of relapse with antigen-loss or antigen-escape variants that occurred in patients that had been treated with CD19 CAR-T or BCMA CAR-T. In the majority of patients, these relapses occurred at a time when CAR-T had contracted and were only present at low frequency. In this situation, the very low-level antigen expression on tumor cells is presumably insufficient for re-igniting the antitumor response^22,26^. It should be pointed out however, that in myeloma patients, expansion of CD19 CAR-T does not singularly depend on stimuli from myeloma cells, but may also be driven by normal B cells.

In summary, our data show that single-molecule-sensitive super-resolution imaging methods such as *d*STORM can be used to establish quantitative expression profiles of surface antigens that are expressed at ultra-low levels, and predicts the ability of antigen-specific CAR-T to eradicate these antigen-positive tumor cells. We are presenting *d*STORM-analysis as a method to complement rather than to replace existing clinical pathology methodologies as, e.g., FC and immunohistochemistry (IHC). Despite advanced microscopy and computing capabilities, *d*STORM-analysis remains very time- and labor-intensive. Using the workflow as presented in this study, the sample preparation, imaging and data analysis of ~10 cells requires 30–60 min, depending on the density of adherent cells in the respective sample. With FC, a substantially higher number of cells can be analyzed in a fraction of this time in semi-automated manner and accordingly, this method will certainly remain a major backbone for detecting target antigens in routine clinical pathology. However, super-resolution microscopy can aid in stratifying patients, e.g., according to CD19 expression in order to identify myeloma patients who have the highest chance to benefit from this novel, innovative treatment. These insights are relevant not only for CD19 CAR-T in myeloma, but also for CAR-T approaches targeting alternative antigens in other hematologic and solid tumor malignancies. Indeed, we show CD20 expression on myeloma cells by *d*STORM and recognition of myeloma cells that express CD20 at low density by CD20 CAR-T.

Our study illustrates the challenge that CAR-T can be more sensitive in detecting antigens on tumor cells than established analytical tools in clinical pathology. Consequently, more sensitive detection methods than FC (and IHC) ought to be implemented into clinical routine in order to guide patient and antigen selection for CAR-T therapy, and in order to detect low-level expression in healthy tissues to prevent toxicity. Efforts to implement *d*STORM-analysis into clinical pathology are ongoing at our institution.

## Methods

### Human subjects

Bone marrow aspirates were obtained from patients with multiple myeloma, and T-cells for CAR-modification were isolated from the peripheral blood of healthy donors. All participants provided written informed consent to participate in research protocols approved by the institutional review board of the University of Würzburg.

### Primary myeloma cells

Freshly aspirated bone marrow was diluted 1:10 in phosphate-buffered saline (PBS), and leukocytes were isolated using Ficoll-hypaque density centrifugation in 50 ml LeukoSep tubes (Greiner Bio One, Frickenhausen, Germany). Myeloma cells were isolated using positive selection with CD138-MicroBeads (Miltenyi, Bergisch-Gladbach, Germany) and used for functional assays next-day.

### Cell lines and cell culture media

NALM-6 (DSMZ, Heidelberg, Germany) and K562 (both ATCC, Manassas, VA, USA) cells were maintained in RPMI-1640 medium containing 8% fetal calf serum (FCS), 2 mM l-glutamine, and 100 U/ml penicillin/streptomycin (all components from Gibco, ThermoScientific, Schwerte, Germany). K562_CD19 cells were generated by lentiviral transduction with human *CD19*. Primary myeloma cells and T-cells were maintained in RPMI-1640 medium containing 8% human serum, 2 mM Glutamax, 0.1% β-mercaptoethanol and 100 U/ml penicillin/streptomycin (T-cell medium; all other components from Gibco). T-cell cultures were supplemented with 50 U/ml IL-2 (Proleukin, Novartis, Basel, Switzerland).

### Generation of CAR-T

The vector design and experimental procedure has been described in a previous study^27^. In brief, peripheral blood mononuclear cells (PBMCs) of healthy donors were purified using Ficoll-hypaque density centrifugation in 50 ml LeukoSep tubes (Greiner Bio One), and CD8^+^ T-cells were isolated using negative magnetic sorting (CD8^+^ T-cell Isolation Kit, human, Miltenyi). T-cells were stimulated with anti-CD3/CD28 magnetic beads (Dynabeads® Human T-Activator CD3/CD28, ThermoScientific) and transduced with an epHIV7 lentivirus encoding a CAR construct comprising the following: an anti-CD19 or -CD20 single chain variable fragment derived from FMC63 or Leu16; an IgG4-Fc hinge spacer; a CD28 transmembrane region; a 4–1BB_CD3ζ signaling module; and a truncated epidermal growth factor receptor (EGFR) transduction marker^28^. T-cells were enriched for EGFRt^+^ using the biotinylated anti-EGFR monoclonal antibody (mAb) Cetuximab (Merck, Darmstadt, Germany) and anti-Biotin Microbeads (Miltenyi). Purified CD19 CAR-T, CD20 CAR-T and non-transduced control T-cells were expanded for 10 days with irradiated CD19^+^/CD20^+^ EBV-LCL feeder cells at a ratio of 1:7 and IL-2 50 IU/ml on days 1, 4, and 7^29^ and stored in aliquots in liquid nitrogen until functional testing.

### Antibodies and flow cytometry

Antibodies against CD19 (clone HIB19, AF647, #302220), CD20 (clone 2H7, AF647, #302318), CD38 (clone HIT2, AF488, # 303512), CD138 (clone MI15, PE, #356504 and unconjugated, #356502) from BioLegend (London, United Kingdom); IFN-γ (clone B27, FITC, # 554700) from BD Biosciences (Heidelberg, Germany), and CD8 (clone BW135/80, VioBlue, # 130–094–152) from Miltenyi as well as 7-AAD to exclude dead cells from analysis were used. For *d*STORM-microscopy, an anti-CD138 antibody was conjugated to AF555 (ThermoFisher Scientific). Flow analyses were performed with a FACS Canto II (BD) machine and analyzed using FlowJo software (TreeStar, Ashland, OR).

### Experimental procedures

CD19 CAR-T, CD20 CAR-T, and non-transduced control T-cells were thawed, washed, and maintained overnight in T-cell medium with low-dose IL-2 (10 IU/ml). Then, 1 × 10^5^ T-cells were co-cultured with 2.5 × 10^4^ primary myeloma cells or control tumor cell lines for 4 h in 96-well round-bottom plates in the absence (for microscopy measurements) or presence of GolgiStop™ (BD). GolgiStop™-treated cells were permeabilized using the Cytofix/Cytoperm Kit (BD) and stained for intracellular IFN-γ (1:20 diluted). For flow cytometric analysis of CD19/CD20 expression, untouched primary myeloma cells were washed and stained with anti-CD38-AF488, anti-CD138-PE, and anti-CD19-AF647, anti-CD20-AF647, or AF647 isotype control (all 1:20 diluted). according to the manufacturer’s instructions and subsequently washed and analyzed. For microscopy measurements, LabTek chamber slides (Nunc™ Lab-Tek™ II Chamber Slide™ System, ThermoFisher Scientific) were coated with poly-d-lysine and primary myeloma cells (or cell lines/co-cultures) and allowed to adhere for 90 min at 37 °C. Afterwards, cells were washed with PBS and stained with anti-CD38-AF488, anti-CD138-AF555 and anti-CD19-AF647, anti-CD20-AF647 or AF647 isotype control (all 1:20 diluted). Cells were washed and fixed with 4% paraformaldehyde and used for dSTORM-analyses.

### *d*STORM imaging

For reversible photoswitching of Alexa Fluor 647, a PBS-based imaging buffer (pH 7.4) was used that contained 80 mM β-mercaptoethylamine (Sigma-Aldrich, Taufkirchen, Germany) and an oxygen scavenger system containing 3% (w/v) glucose, 4 U/ml glucose oxidase and 80 U/ml catalase. *d*STORM measurements were performed as previously described^12,13^: we used an Olympus IX-71 inverted microscope (Olympus, Hamburg, Germany) equipped with an oil-immersion objective (APON 60XOTIRF, Olympus) and a nosepiece stage (IX2-NPS, Olympus). AF647, AF555, and AF488 were excited with the appropriate laser systems (Genesis MX 639 and MX 561 from Coherent, Göttingen, Germany; iBeam smart 488 nm, Toptica, Gräfelfing, Germany). The excitation light was spectrally cleaned by appropriate bandpass filters and then focused onto the backfocal plane of the objective. To switch between different illumination modes (epi and TIRF illumination), the lens system and mirror were arranged on a linear translation stage. A polychromatic mirror (HC 410/504/582/669, Semrock, Rochester, NY, USA) was used to separate excitation (laser) and emitted (fluorescent) light. The fluorescence emission was collected by the same objective and transmitted by the dichroic beam splitter and several detection filters (HC 440/521/607/700, Semrock; HC 679/41, Semrock, for Alexa 647; HQ 610/75, Chroma (Bellows Falls, VT, USA), for Alexa 555; ET 525/50, Chroma, for Alexa 488), before being projected onto two electron-multiplying CCD cameras (both iXon Ultra 897, Andor, Belfast, UK; beam splitter 635 LP, Semrock). A final pixel size of 128 nm was generated by placing additional lenses in the detection path. Excitation intensity was ~3.3 kW/cm^2^. Typically, 15,000 frames were recorded with a frame rate of ~67 Hz (15 ms exposure time).

### Image reconstruction and data analysis

From the recorded image stack, a table with all localizations as well as a reconstructed *d*STORM image was generated using the single-molecule localization software rapidSTORM 3.3^30^. Only CD38/CD138 double-positive cells (i.e., myeloma cells) were further analyzed for CD19/CD20 expression. Quantification of CD19 and CD20 was performed with a custom-written Mathematica (Wolfram Research, Inc., Mathematica, Version 11.0, Champaign, IL, USA) script. The analysis routine included the following steps: fluorescent spots containing <800 photons per frame were discarded. Repeated localizations coming from one antibody were grouped using an alpha-shape algorithm with an alpha value of 30. To approximate a useful alpha value, which allows to determine the exact number of localizations detected per antibody, the alpha value was varied over a large range and plotted against the number of localizations per cluster, the cluster density and the clustered localizations, respectively (Supplementary Fig. 9). At an alpha value of 30, the cluster density reaches a maximum and the number of localizations per cluster saturated in a plateau, which both indicate that the antigens are evenly distributed and separated from each other with distances larger than the cluster sizes (Supplementary Fig. 9A). This also confirmed that the overall density of detected antibodies was small enough to yield well-separated alpha-shapes. Antibody densities (CD19, CD20 or isotype) were calculated from the number of grouped localizations divided by the area of the bottom plasma membrane of each cell, as determined with a region of interest (ROI)-selector. A total of 10–80 cells per patient and condition were analyzed to obtain CD19, CD20, and isotype antibody density distributions. To distinguish between non-specific (negative subpopulation) and specific (positive subpopulation) binding of CD19/CD20 antibodies, detected antibody density distributions were fitted to a one- or two-component log-normal distribution. Relative contributions of non-specifically and specifically bound antibodies were estimated, together with the average density (localizations µm^−2^) of specifically bound antibodies. The significance of CD19 distribution estimates was statistically tested using an Anderson-Darling test (rejected for *p*-values < 0.05). To determine the number of antigens (CD19/CD20) per cell, the average cell surface area of 20–50 cells per patient was estimated by measuring the diameter of each cell using transmitted light microscopy and calculating the cell surface area assuming a spherical cell shape. For comparing the density variations between the different patients, we computed the mean values of the non-logarithmized sample values from all cells for each patient. An uncertainty estimate for these mean values (shown as error bars in Fig. 2d) was provided by simulation. The error was computed by generating a random sample of size equal to the number of observed cells that follows the fitted log-normal distribution for each patient. We computed the mean value for this random sample, repeated the procedure 1000 times and showed the standard deviation of all means as error bar.

### Reporting summary

Further information on research design is available in the Nature Research Reporting Summary linked to this article.

## Supplementary information

Supplementary Information

Reporting Summary

Source Data

## Data Availability

All data that support the findings described in this study are available within the manuscript and the related supplementary information. The data supporting Fig. 2d are available as a Source data file. Any other data is available from the corresponding authors upon reasonable request.

## Code availability

Custom Mathematica code for analysis procedures can be made available upon reasonable request to the corresponding author.
