# Supplementary material for: Super-resolution microscopy reveals ultra-low CD19 expression on myeloma cells that triggers elimination by CD19 CAR-T
Source: Nat Commun. 2019 Jul 17;10:3137. doi: 10.1038/s41467-019-10948-w (PMC6637169; doi:10.1038/s41467-019-10948-w)
Supplement: Supplementary file 1 — Supplementary Information [file 41467_2019_10948_MOESM1_ESM.pdf]

Super-resolution microscopy reveals ultra-low CD19 expression on myeloma cells that triggers elimination by CD19 CAR-T

Nerreter et al

SUPPLEMENTARY INFORMATION

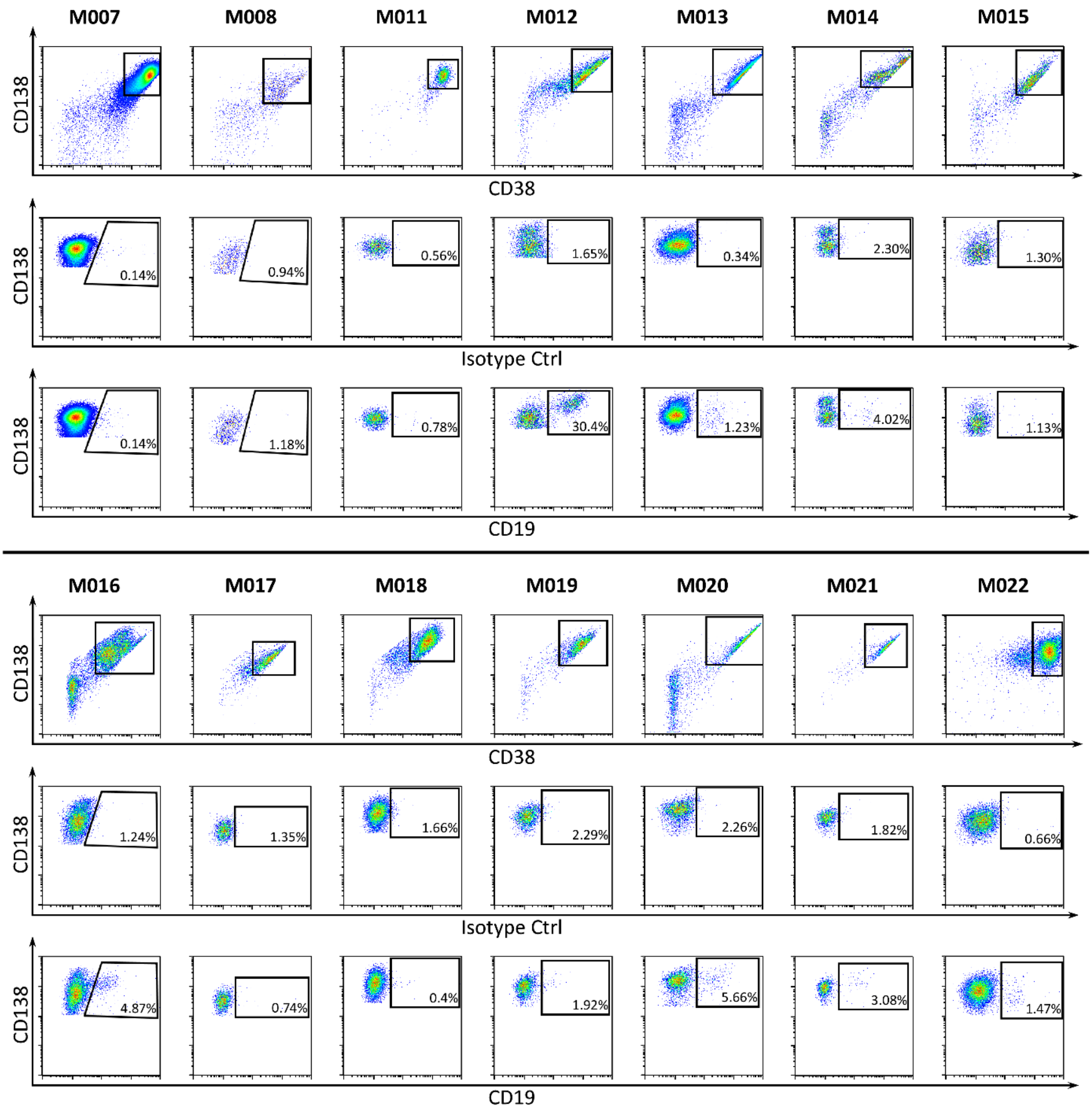

Supplementary Figure 1. Detection of CD19 on myeloma cells by flow cytometry. CD138-purified bone marrow aspirates from multiple myeloma patients were stained with antibodies against CD138 and CD38 to detect myeloma cells (first line) and a CD19-specific antibody (third line) or a corresponding isotype control (second line) and measured by flow cytometry. Gates were set on plasma cells (FSC/SSC), 7-AAD$^-$ and CD138$^+$/CD38$^+$ myeloma cells. Percentages indicated refer to CD19-positive cells within the CD138$^+$/CD38$^+$ subset.

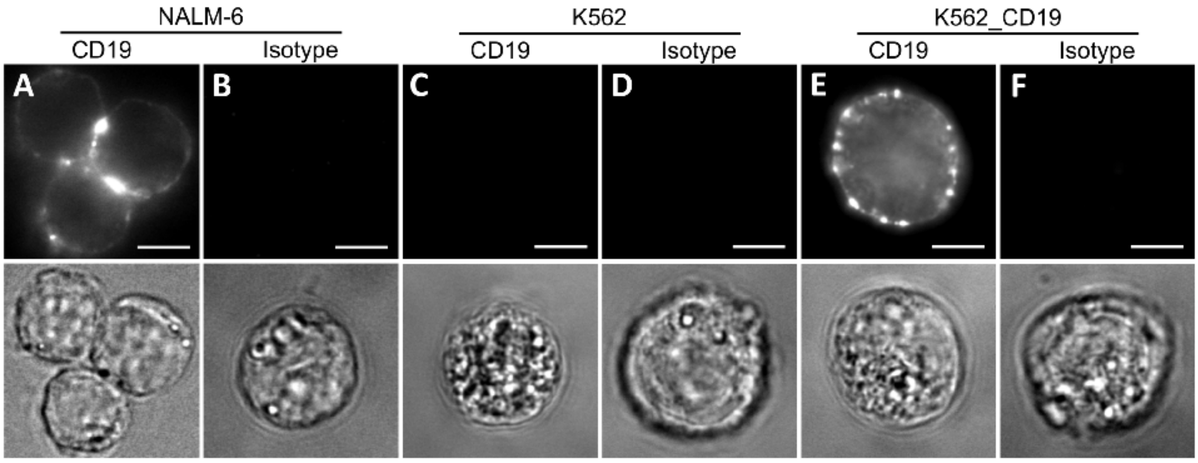

Supplementary Figure 2. Specificity of anti-CD19 antibody. The anti-CD19 antibody employed in this study was tested for binding specificity by conventional wide-field microscopy (*upper rows:* normalized fluorescence, *bottom rows:* transmitted light). NALM-6 (**A**, **B**), K562 (**C, D**) and CD19 expressing K562_CD19 cells (**E, F**) were stained with anti-CD19-AF647 antibody (*column label:* CD19) and its corresponding isotype-AF647 antibody (*column label:* Isotype). Scale bars, 7 μm.

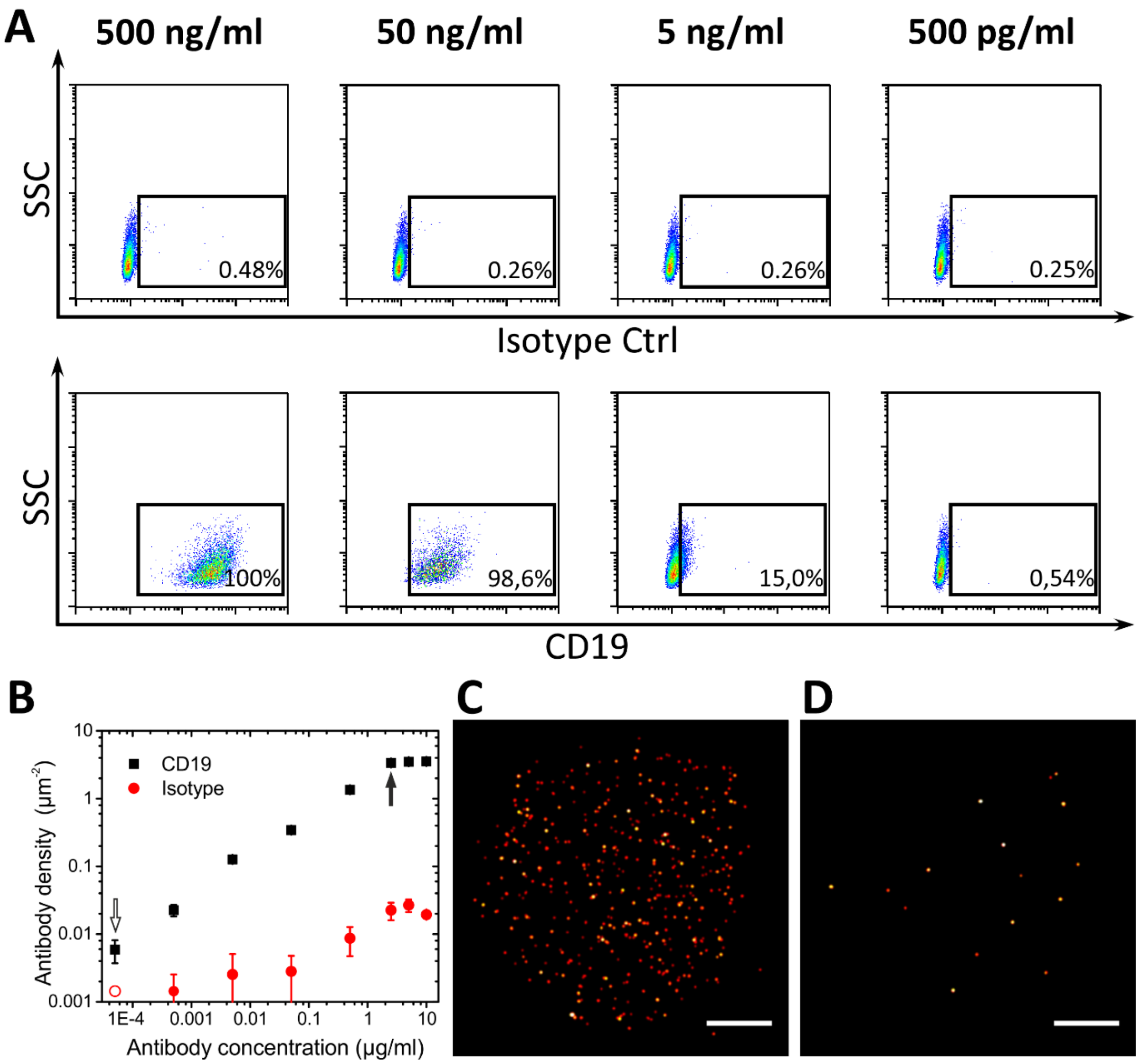

Supplementary Figure 3. *d*STORM is 1000-times more sensitive than flow cytometry in detecting CD19. The $CD38^+/CD138^+/CD19^+$ ALL cell line NALM-6 was stained with antibodies against CD138, CD38 and CD19 or the corresponding isotype control. (**A**) Flow cytometric detection of CD19 on NALM-6 cells with decreasing dilutions of CD19-specific antibody (lower row) or corresponding isotype control (upper row). (**B**) Detection of CD19 antibody (black squares) and isotype control (red circles) by *d*STORM. At a CD19 antibody concentration of 2.5 µg/ml (1:20 dilution), the CD19 density saturated at 3.4 ± 0.2 (SEM, Standard Error of the Mean) CD19 antibodies/μm^2 (filled arrow). The lowest detectable density was 0.006 ± 0.002 CD19 antibodies/μm^2, which was at 50 pg/ml (1:10^6 dilution, open arrow). At an isotype antibody concentration of 50 pg/ml, it was not possible to detect any molecules (0 molecules/μm^2), which is represented as a red open circle in the graph. Corresponding *d*STORM images are depicted in (**C**), 2.5 µg/ml, and (**D**), 50 pg/ml CD19 antibody. Scale bars, 2 µm.

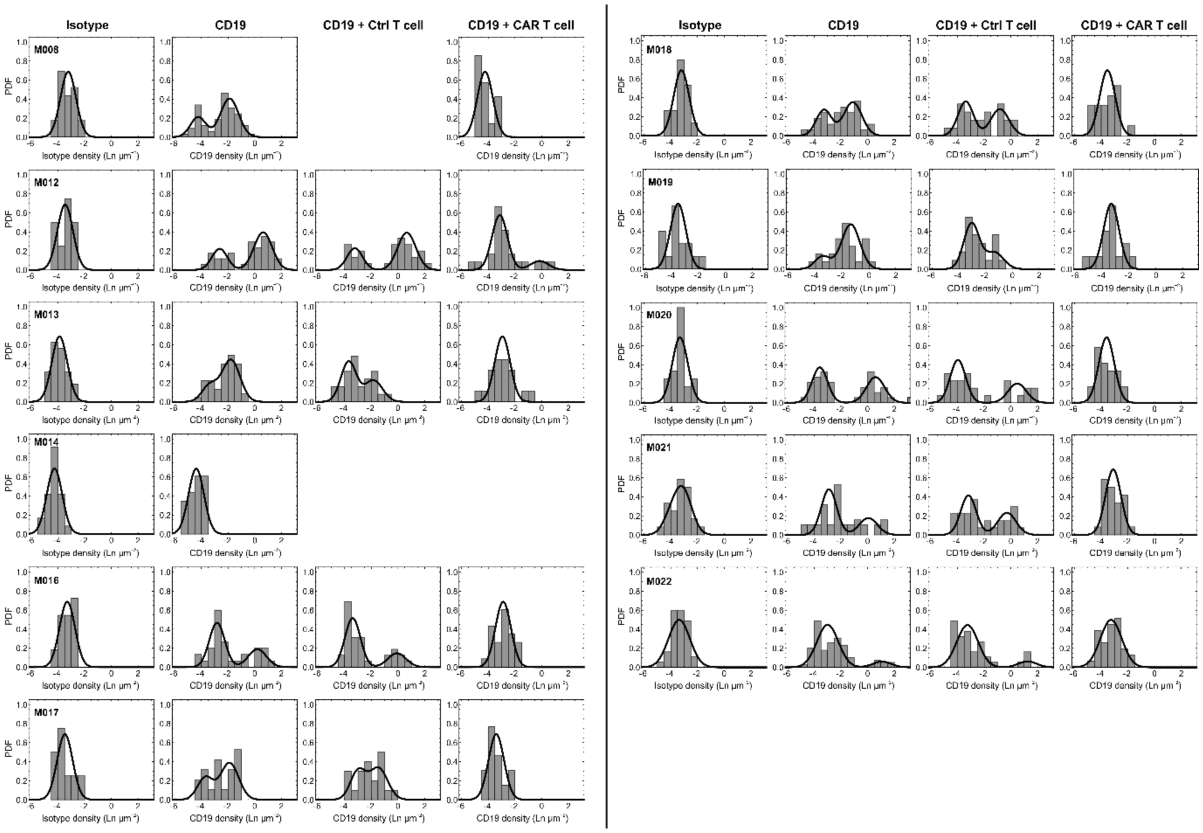

Supplementary Figure 4. Quantification of CD19 on myeloma cells by *d*STORM and elimination of CD19-positive myeloma cells by CD19 CAR-T. CD138-purified bone marrow aspirates from multiple myeloma patients were stained with antibodies against CD138 and CD38 to detect myeloma cells and a CD19-specific antibody or corresponding isotype control as indicated. Shown are distributions of all CD19-positive patients and one representative negative patient (M014). Left panels: Logarithmic number (natural logarithm, Ln) of isotype and CD19 antibodies per µm^2 of untreated myeloma cells. Right panels: Logarithmic CD19 densities of control T-cell- and CAR-T-cell-treated myeloma cells. Density distributions were subsequently divided into a CD19-positive subpopulation (CD19-positive cells) and a CD19-negative subpopulation (CD19-negative cells). The latter group was defined by the density distribution pattern of the isotype control antibody (non-specific binding of the control antibody to the plasma membrane and glass surface). Distributions were fitted with a one or two component log-normal function that was dependent on the fit accuracy calculated with an Anderson-Darling test (rejected at a p-value < 0.05). Effect of control T-cells was not evaluated for patient M008. M014 is an example of a completely CD19^{-} patient. PDF: probability density function. Data are also summarized in Table 1.

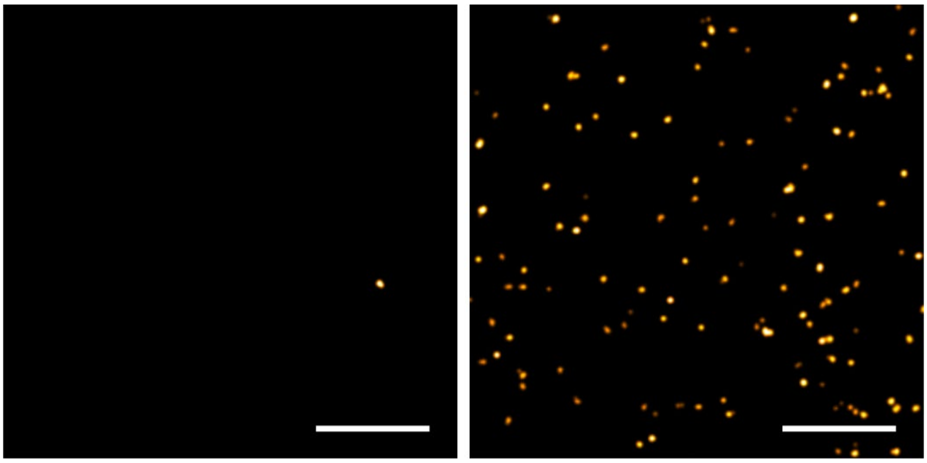

Supplementary Figure 5. CD19^{low} and CD19^{high} expression on primary myeloma cells. 4×4 μm sections of reconstructed *d*STORM images showing single CD19 molecules on the basal plasma membrane of immobilized multiple myeloma cells. (**A**) Low CD19 expression (~ 13 molecules/cell, M017) and (**B**) high CD19 expression (~ 3,000 molecules/cell, M022). Scale bars, 1 μm.

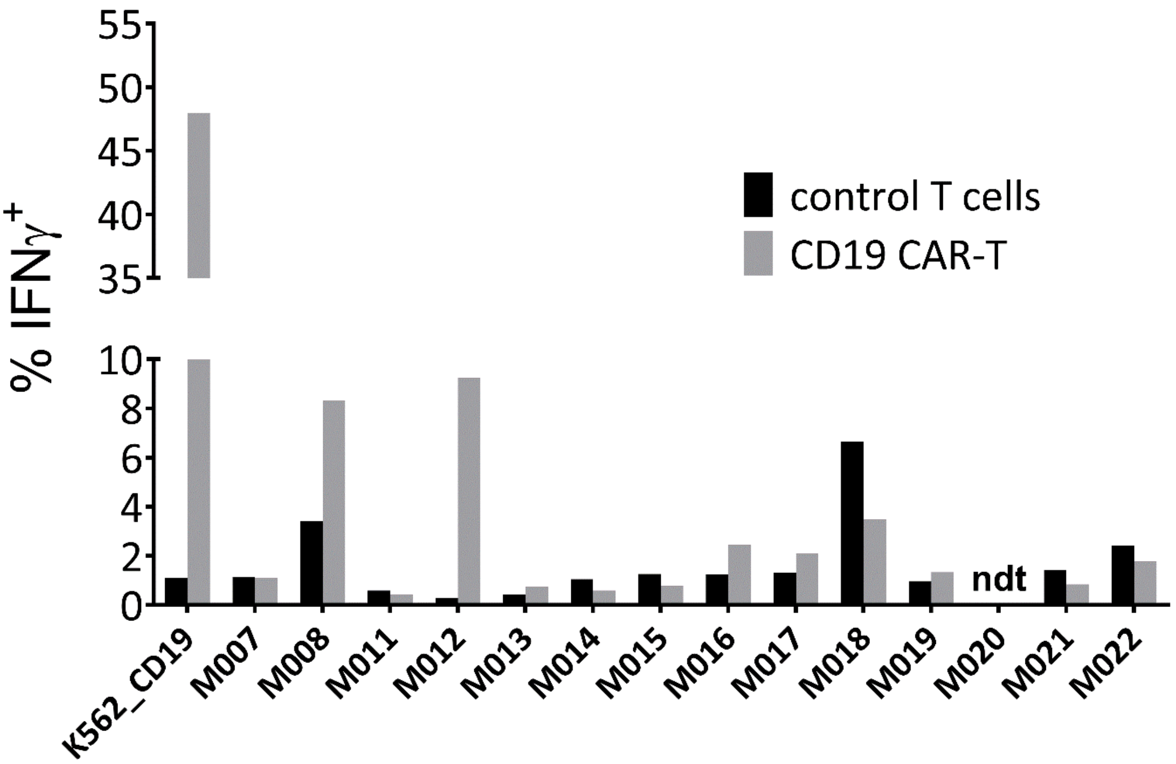

Supplementary Figure 6. IFNγ production by CD19 CAR-T upon coculture with myeloma cells. CD19 CAR-T (light gray) or untransduced control CD8$^+$ T-cells (black) were cocultured with primary myeloma cells or K562_CD19 at an effector:target ratio of 4:1 for 4 h in the presence of GolgiStop™. T-cells were treated with Cytofix/Cytoperm and stained with anti-CD8 and anti-IFNγ mAbs. Shown is the percentage of IFNγ$^+$ T-cells in the presence of primary myeloma or K562_CD19 cells minus the percentage of IFNγ$^+$ T-cells cultured for 4 h with medium only. Gates were set on lymphocytes (FSC/SSC), CD8$^+$ and IFNγ$^+$ cells. Every column represents a single experiment, except for K562_CD19 (n=12). ndt: cytokine production was not assessed for patient M020.

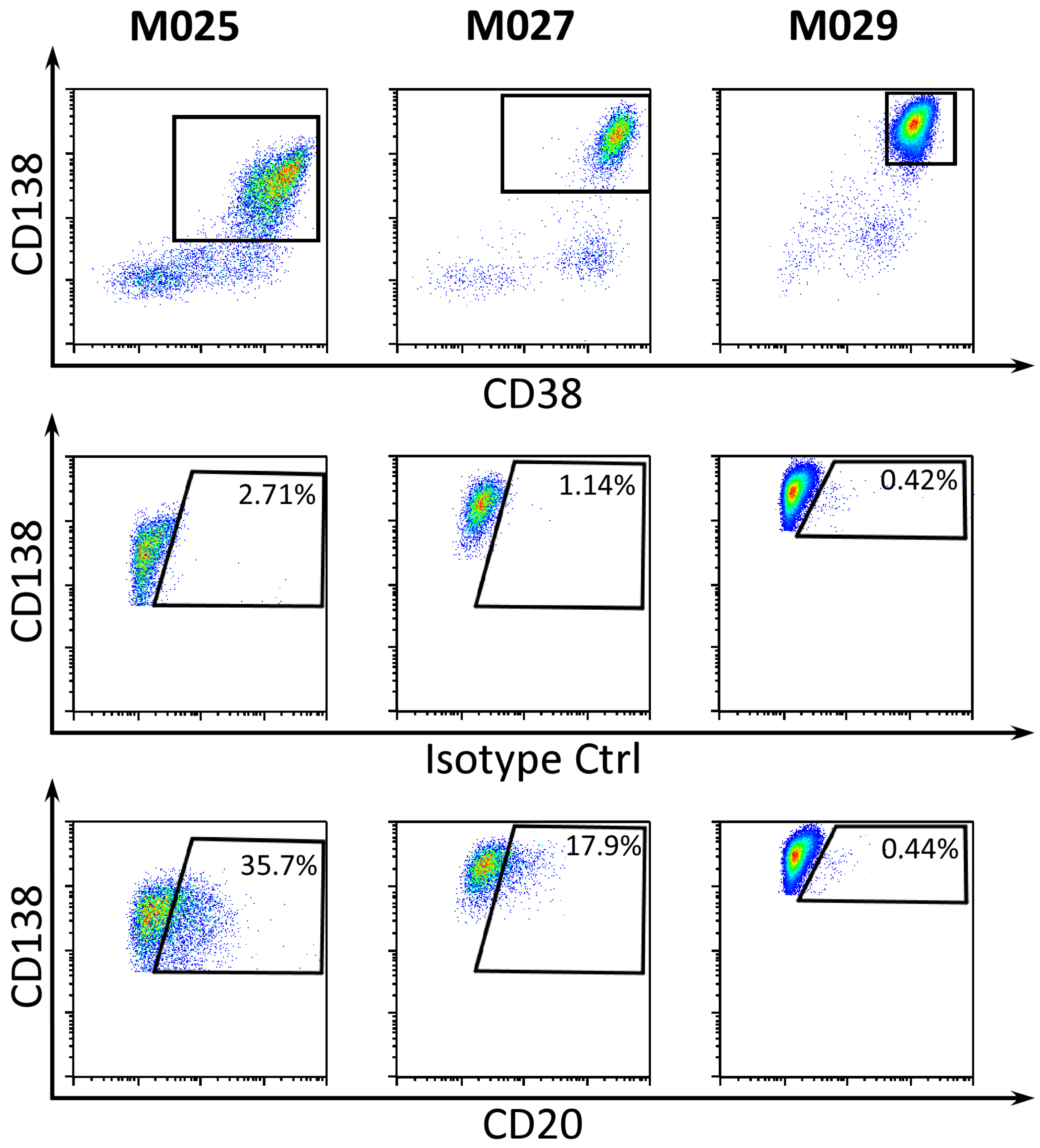

Supplementary Figure 7. Detection of CD20 on myeloma cells by flow cytometry. Flow cytometric analysis of CD20-expression on primary myeloma cells purified from bone marrow aspirates. Gating strategy for dot plots shown: FSC/SSC plasma cell gate → 7-AAD$^-$ → CD138$^+$/CD38$^+$→ Isotype control or CD20.

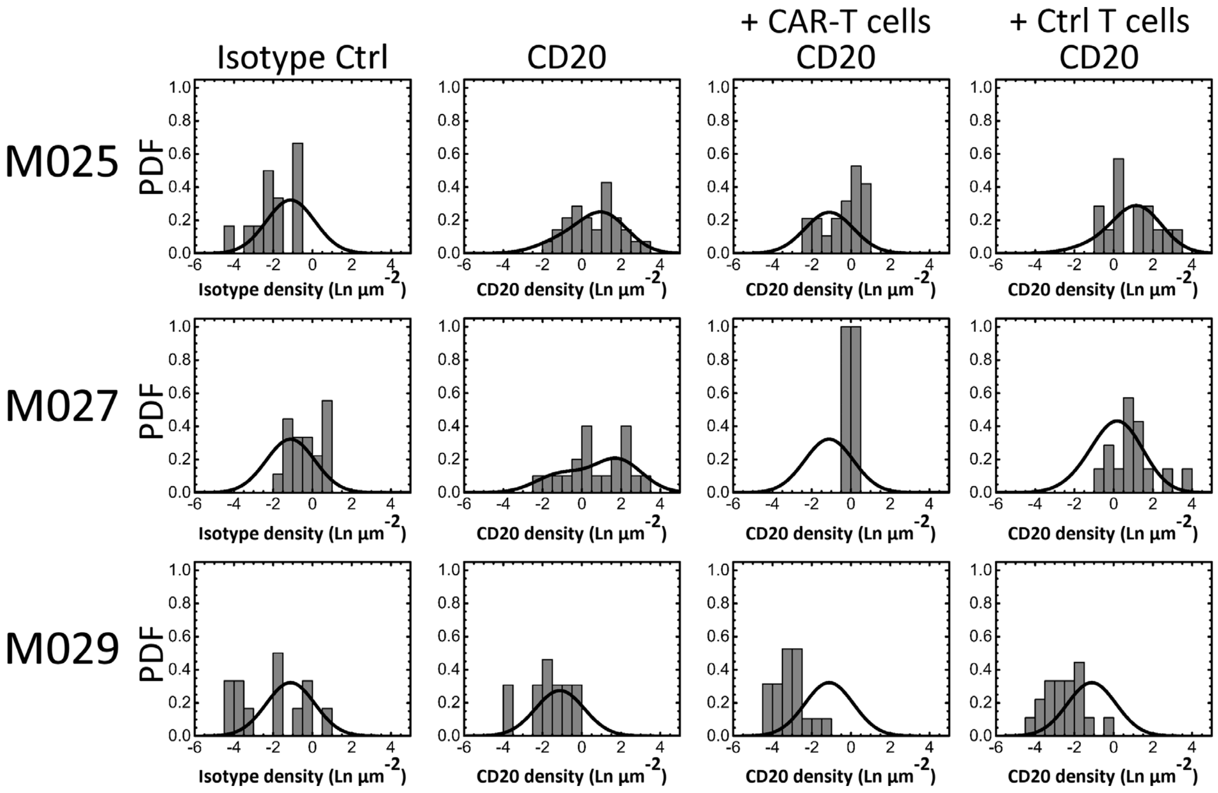

Supplementary Figure 8. Quantification of CD20 on myeloma cells by *d*STORM and elimination of CD20-positive myeloma cells by CD20 CAR-T. CD138-purified bone marrow aspirates from 3 multiple myeloma patients were stained with antibodies against CD138 and CD38 to detect myeloma cells and a CD20-specific antibody or corresponding isotype control as indicated. Left panels: Logarithmic number (natural logarithm, Ln) of isotype and CD20 antibodies per μm^2 of untreated myeloma cells. Right panels: Logarithmic CD20 densities of control T-cell- and CAR-T-cell-treated myeloma cells. Density distributions were subsequently divided into a CD20-positive subpopulation (CD20-positive cells) and a CD20-negative subpopulation (CD20-negative cells). The latter group was defined by the density distribution pattern of the isotype control antibody (non-specific binding of the control antibody to the plasma membrane and glass surface). Distributions were fitted with a one or two component log-normal function. PDF: probability density function. Data are also summarized in Supplementary Table 3.

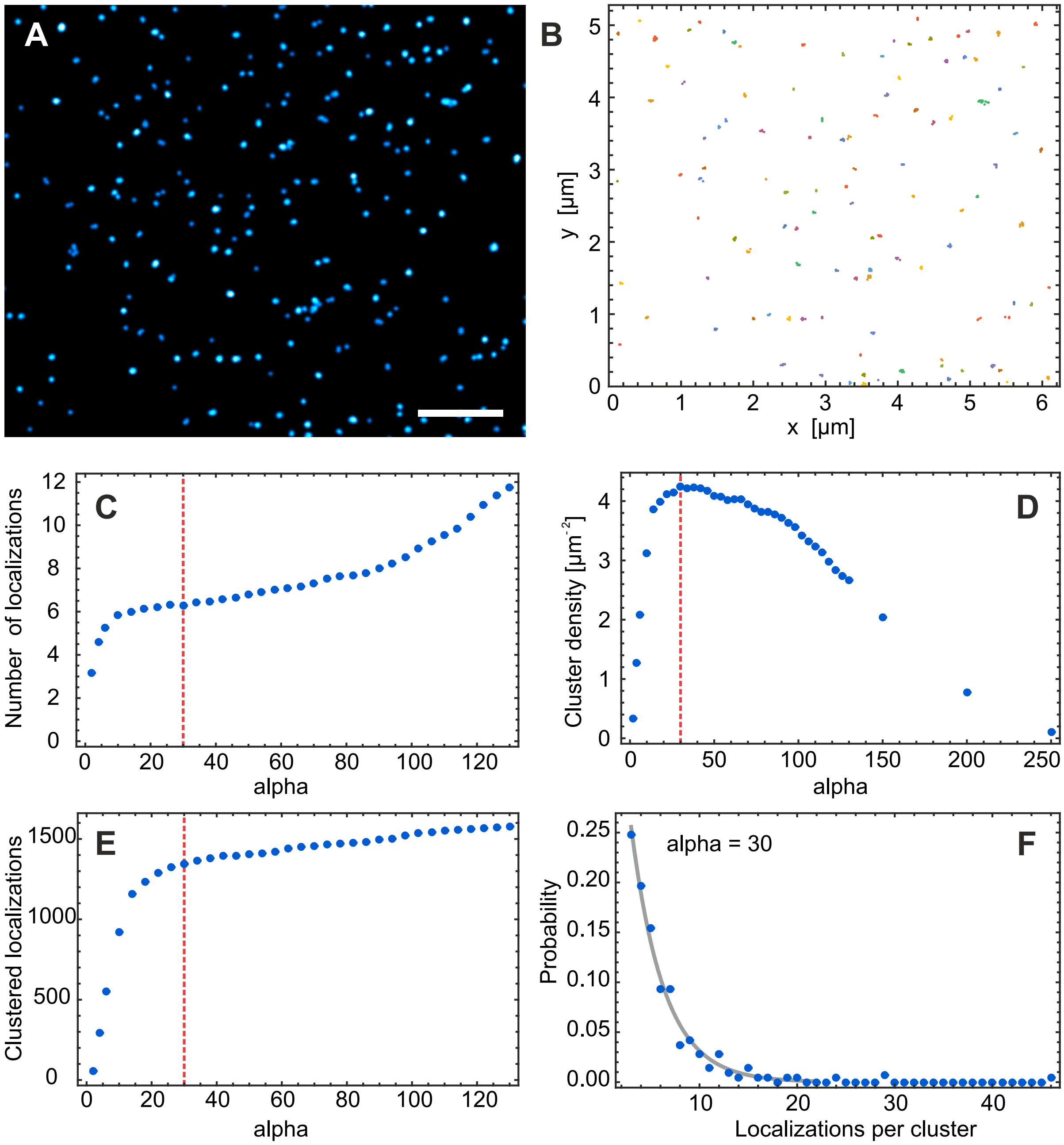

Supplementary Figure 9. Clustering CD19 molecules on myeloma cells using alpha shapes. (**A**) Section of a reconstructed *d*STORM image of CD19 molecules stained with anti-human CD19 Alexa Fluor 647 antibody. (**B**) Correspondent alpha shape diagram illustrating the obtained clusters in different colors. Repeated localizations coming from single fluorescent spots were clustered with alpha shapes and an alpha value of 30 nm. (**C**–**E**) A suitable alpha value was determined by serial cluster finding, i.e. varying α from small to large values which influences the number of localizations per cluster (**C**), cluster density (**D**) and the number of clustered localizations (**E**) (non-clustered localizations were discarded). Red dotted lines mark positions at α = 30 nm. (**F**) Relative distribution of the number of localizations per cluster for α = 30 nm (mean ≈ 6.3 localizations per cluster). Scale bar, 1 µm.

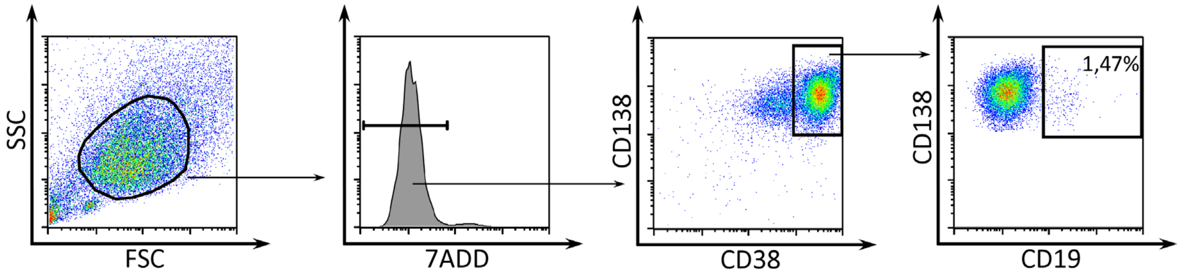

Supplementary Figure 10. Gating strategy used in FC experiments. Flow cytometric analysis of CD19-expression on primary myeloma cells as depicted in **Figure 1**. Purified bone marrow aspirates were analyzed and a plasma cell gate was set (FSC/SSC) -> viable cells (7-AAD$^-$)-> Multiple Myeloma cells (CD138$^+$/CD38$^+$). Expression of CD20 was similarly analyzed (**Figure 4**)

Supplementary Table 1: Patient characteristics

Characteristic	all patients (n=14)
Median age (range) - yr	62.3 (52 – 81)
Male - no. (%)	7 (50)
Salmon & Durie* stage at diagnosis - no. (%)	
I	3 (21)
II	1 (7)
IIIA	8 (57)
IIIB	2 (14)
Myeloma subtype - no. (%)	
IgG	8 (57)
IgA	1 (7)
IgD	1 (7)
Light chain	4 (29)
Cytogenetic profile** - no. (%)	
High-risk	5 (36)
Standard risk	9 (64)
Time from diagnosis (range) - months	16 (0 – 69)
Remission state*** - no. (%)	
Primary diagnosis	4 (29)
Very good partial response	2 (14)
Progressive disease	8 (57)
Bone marrow infiltration - % (range)	25 (10 – 99)
Previous therapy regimens	
Median no. (range)	1 (0 – 3)
Previous therapies - no. (%)	
Hematopoietic stem-cell	9 (64)
Lenalidomide	6 (43)
Proteasome inhibitor	10 (71)

* A clinical staging system for multiple myeloma based on the correlation of the measured myeloma cell mass with presenting clinical features, response to treatment, and survival[1].
** Cytogenetic analysis: a high-risk cytogenetic profile refers to adverse FISH including IgH translocations (t(4;14) or t(14;16) or t(14;20)), 17p13 del and/or 1q21 gain[2].
*** International Myeloma Working Group consensus criteria for response and minimal residual disease assessment in multiple myeloma[3].

Supplementary Table 2: Antibody titration on NALM-6 cells

Antibody	Molecules per cell	
	**Anti-CD19**	**Isotype**
10 µg/ml	1880 ± 220	72 ± 20
5 µg/ml	1860 ± 180	85 ± 25
2.5 µg/ml	1780 ± 210	50 ± 30
500 ng/ml	710 ± 110	44 ± 28
50 ng/ml	182 ± 29	7.1 ± 6.4
5 ng/ml	67 ± 10	1.3 ± 1.4
500 pg/ml	12.0 ± 2.9	0.8 ± 0.6
50 pg/ml	3.1 ± 1.3	0 ± 0
0	0 ± 0	0 ± 0

±: SEM (standard error of the Mean)

Supplementary Table 3 – Summary of data obtained by *d*STORM and flow cytometry

#	ID	Flow Cytometry Δ% CD20$^+$ (% anti-CD20 - % isotype)	*d*STORM % CD20$^+$	CD20 molecules/cell* (range)	Elimination by CD20 CAR-T
15	M025	33 (35.7 – 2.7)	76.7	650 (55 – 7,724)	+
17	M027	16.8 (17.9 – 1.1)	64.7	1,770 (149 – 21,045)	+
18	M029	0 (0.42 – 0.44)	0	0	0

(*) Mean, in brackets: Calculated data ranging from small ($\exp(\mu-2\sigma)$) to high ($\exp(\mu+2\sigma)$) values (95.45% of all values lie within this range).
Δ % CD20$^+$: the percentage of the cells in the CD20-positive gate for the isotype control was subtracted from the percentage of cells in the CD20-positive gate for the respective CD20 staining.

References

1. Durie, B.G. & Salmon, S.E. A clinical staging system for multiple myeloma. Correlation of measured myeloma cell mass with presenting clinical features, response to treatment, and survival. *Cancer* **36**, 842-854 (1975).
2. Chng, W.J., *et al.* IMWG consensus on risk stratification in multiple myeloma. *Leukemia* **28**, 269-277 (2014).
3. Kumar, S., *et al.* International Myeloma Working Group consensus criteria for response and minimal residual disease assessment in multiple myeloma. *Lancet Oncol* **17**, e328-e346 (2016).
