# Supplementary material for: Super-resolution microscopy reveals ultra-low CD19 expression on myeloma cells that triggers elimination by CD19 CAR-T
Source: Nat Commun. 2019 Jul 17;10:3137. doi: 10.1038/s41467-019-10948-w (PMC6637169; doi:10.1038/s41467-019-10948-w)
Supplement: Supplementary file 2 — Reporting Summary [file 41467_2019_10948_MOESM2_ESM.pdf]

nature research

Corresponding author(s): Markus Sauer & Michael Hudecek

Last updated by author(s): May 28, 2019

Reporting Summary

Nature Research wishes to improve the reproducibility of the work that we publish. This form provides structure for consistency and transparency in reporting. For further information on Nature Research policies, see Authors & Referees and the Editorial Policy Checklist.

Statistics

For all statistical analyses, confirm that the following items are present in the figure legend, table legend, main text, or Methods section.

n/a	Confirmed	
☐	☒	The exact sample size (*n*) for each experimental group/condition, given as a discrete number and unit of measurement
☐	☒	A statement on whether measurements were taken from distinct samples or whether the same sample was measured repeatedly
☐	☒	The statistical test(s) used AND whether they are one- or two-sided<br>*Only common tests should be described solely by name; describe more complex techniques in the Methods section.*
☐	☒	A description of all covariates tested
☐	☒	A description of any assumptions or corrections, such as tests of normality and adjustment for multiple comparisons
☐	☒	A full description of the statistical parameters including central tendency (e.g. means) or other basic estimates (e.g. regression coefficient) AND variation (e.g. standard deviation) or associated estimates of uncertainty (e.g. confidence intervals)
☒	☐	For null hypothesis testing, the test statistic (e.g. *F*, *t*, *r*) with confidence intervals, effect sizes, degrees of freedom and *P* value noted<br>*Give P values as exact values whenever suitable.*
☒	☐	For Bayesian analysis, information on the choice of priors and Markov chain Monte Carlo settings
☒	☐	For hierarchical and complex designs, identification of the appropriate level for tests and full reporting of outcomes
☒	☐	Estimates of effect sizes (e.g. Cohen's *d*, Pearson's *r*), indicating how they were calculated

Our web collection on statistics for biologists contains articles on many of the points above.

Software and code

Policy information about availability of computer code

Data collection	BD FACS Diva 8.0; rapidSTORM 3.3.
Data analysis	GraphPad Prism 6; FlowJo 10.4.2; rapidSTORM 3.3; Wolfram Mathematica 11.2.

For manuscripts utilizing custom algorithms or software that are central to the research but not yet described in published literature, software must be made available to editors/reviewers. We strongly encourage code deposition in a community repository (e.g. GitHub). See the Nature Research guidelines for submitting code & software for further information.

Data

Policy information about availability of data

All manuscripts must include a data availability statement. This statement should provide the following information, where applicable:
- Accession codes, unique identifiers, or web links for publicly available datasets
- A list of figures that have associated raw data
- A description of any restrictions on data availability

The datasets generated during and/or analysed during the current study are available from the corresponding author on reasonable request.

Field-specific reporting

Please select the one below that is the best fit for your research. If you are not sure, read the appropriate sections before making your selection.

☒ Life sciences ☐ Behavioural & social sciences ☐ Ecological, evolutionary & environmental sciences

For a reference copy of the document with all sections, see nature.com/documents/nr-reporting-summary-flat.pdf

Life sciences study design

All studies must disclose on these points even when the disclosure is negative.

Sample size	Sample sizes were determined based on the estimates from preliminary experiments and similar studies in the previous manuscripts so that reasonable statistical analyses could be conducted.
Data exclusions	No data was excluded.
Replication	Experimental findings reported in this manuscript were reliably reproduced.
Randomization	Randomization was not relevant to this study as CD19/CD20 status of material was not know in advance.
Blinding	Blinding was not relevant to this study as CD19/CD20 status of material was not know in advance.

Reporting for specific materials, systems and methods

We require information from authors about some types of materials, experimental systems and methods used in many studies. Here, indicate whether each material, system or method listed is relevant to your study. If you are not sure if a list item applies to your research, read the appropriate section before selecting a response.

Materials & experimental systems

n/a	Involved in the study	
☐	☒	Antibodies
☐	☒	Eukaryotic cell lines
☒	☐	Palaeontology
☒	☐	Animals and other organisms
☐	☒	Human research participants
☒	☐	Clinical data

Methods

n/a	Involved in the study	
☒	☐	ChIP-seq
☐	☒	Flow cytometry
☒	☐	MRI-based neuroimaging

Antibodies

Antibodies used	CD19 (clone HIB19, AF647), CD20 (clone 2H7, AF647), CD38 (clone HIT2, AF488), CD138 (clone MI15, PE and unconjugated) from BioLegend (London, United Kingdom); IFN-γ (clone B27, FITC) from BD Biosciences (Heidelberg, Germany), and CD8 (clone BW135/80, VioBlue) from Miltenyi.
Validation	All antibodies are validated by the respective vendors.

Eukaryotic cell lines

Policy information about cell lines

Cell line source(s)	NALM-6 (DSMZ, Heidelberg, Germany), MM.1S and K562 (both ATCC, Manassas, VA, USA).
Authentication	None of the cell lines used in this study were authenticated in our lab, as they were directly purchased from the internationally credible vendors.
Mycoplasma contamination	In our laboratory, the contamination of mycoplasma was regularly examined by PCR, and found no contamination was detected while we conducted experiments concerning this work.
Commonly misidentified lines (See ICLAC register)	No commonly misidentified cell lines were used.

Human research participants

Policy information about studies involving human research participants

Population characteristics	See Supplementary Table 1.
Recruitment	Bone marrow from 17 consecutive patients from our department with MM that had measurable disease by histopathology.
Ethics oversight	Ethics Committee of the University of Würzburg.

Note that full information on the approval of the study protocol must also be provided in the manuscript.

Flow Cytometry

Plots

Confirm that:

☒ The axis labels state the marker and fluorochrome used (e.g. CD4-FITC).

☒ The axis scales are clearly visible. Include numbers along axes only for bottom left plot of group (a 'group' is an analysis of identical markers).

☒ All plots are contour plots with outliers or pseudocolor plots.

☒ A numerical value for number of cells or percentage (with statistics) is provided.

Methodology

Sample preparation	CD19- and CD20-CART and non-transduced control T-cells were thawed, washed and maintained overnight in T-cell medium with low-dose IL-2 (10 IU/mL). Then, 1x105 T-cells were co-cultured with 2.5×104 primary myeloma cells or control tumor cell lines for 4 h in 96-well round-bottom plates in the absence (for microscopy measurements) or presence of GolgiStop™ (BD). GolgiStop™-treated cells were permeabilized using the Cytofix/Cytoperm Kit (BD) and stained for intracellular IFN-γ. For flow cytometric analysis of CD19/CD20 expression, untouched primary myeloma cells were washed and stained with anti-CD38-AF488, anti-CD138-PE and anti-CD19-AF647/anti-CD20-AF647 or AF647 isotype control according to the manufacturer's instructions and subsequently washed and analyzed. For microscopy measurements, LabTek chamber slides (Nunc™ Lab-Tek™ II Chamber Slide™ System, ThermoFisher Scientific) were coated with poly-D-lysine and primary myeloma cells (or cell lines / co-cultures) and allowed to adhere for 90 min at 37°C. Afterwards, cells were washed with PBS and stained with anti-CD38-AF488, anti-CD138-AF555 and anti-CD19-AF647/anti-CD20-AF647 or AF647 isotype control. Cells were washed and fixed with 4% paraformaldehyde and used for dSTORM-analyses.
Instrument	FACS Canto II (BD).
Software	Flow cytmetry data were analyzed by using FlowJo software (FlowJo, LLC.).
Cell population abundance	The purity was confirmed by flow cytometry.
Gating strategy	FSC/SSC plasma cell gate ->7-AAD- -> CD138+/CD38+. See Supplementary Figure 10.

☒ Tick this box to confirm that a figure exemplifying the gating strategy is provided in the Supplementary Information.
